# Diacerein inhibits the pro-atherogenic & pro-inflammatory effects of IL-1 on human keratinocytes & endothelial cells

**DOI:** 10.1371/journal.pone.0173981

**Published:** 2017-03-21

**Authors:** Girish C. Mohan, Huayi Zhang, Lei Bao, Benjamin Many, Lawrence S. Chan

**Affiliations:** 1 Department of Dermatology, University of Illinois at Chicago (UIC)—College of Medicine, Chicago, IL, United States of America; 2 Department of Medicine, Jesse Brown Veterans Affairs Hospital, Chicago, IL, United States of America; 3 Department of Medicine, Capt. James A. Lovell FHCC, North Chicago, IL, United States of America; University of Edinburgh, UNITED KINGDOM

## Abstract

We investigated IL-1-induced regulation of genes related to inflammation and atherogenesis in human keratinocytes and endothelial cells, and if ‘diacerein’, an oral IL-1 inhibiting drug currently approved for use in osteoarthritis, would reverse IL-1’s effects on these cells. Primary human keratinocytes and coronary artery endothelial cells were treated with either IL-1α or IL-1β, with and without diacerein. Using PCR-array, we assessed differential gene-expression regulated by IL-1 and diacerein. We identified 34 pro-atherogenic genes in endothelial cells and 68 pro-inflammatory genes in keratinocytes significantly (p<0.05) regulated at least 2-fold by IL-1, in comparison to control. Diacerein completely or partially reversed this regulation on almost all genes. Using ELISA, we confirmed diacerein’s ability to reverse IL-1-driven gene-regulation of 11 selected factors, at the protein level. The results support a novel idea that diacerein acts as an inhibitor of the pro-atherogenic and pro-inflammatory effects of IL-1. Diacerein may have therapeutic applications to diminish IL-1-induced skin inflammation in psoriasis and attenuate IL-1-induced development of atherosclerosis. Further investigation into diacerein’s effect on skin inflammation, atherogenesis and cardiovascular risk in animal models or humans is warranted.

## Introduction

Psoriasis, a chronic inflammatory skin disease, affects up to 8.5% of adult populations [1]. Psoriasis was previously considered a solely cutaneous entity but recent studies, including a meta-analysis, have shown that moderate-to-severe psoriasis patients have increased cardiovascular risk, with more frequent atherosclerotic events (ex. myocardial infarction) and life expectancy reduction by up to 4 years [2–4]. Although one study showed no link between psoriasis and cardiovascular morbidity [5], data in the literature predominantly demonstrates that an association exists.

Interleukin-1alpha (IL-1α) [6, 7] and interleukin-1beta (IL-1β) [8], the two major subunits of IL-1, are implicated in psoriasis pathogenesis. Increased expression of IL-1α and IL-1β have been found in psoriatic lesional skin in mouse models and in human subjects, as these cytokines directly contribute to the inflammation present in the skin [6, 8, 9]. However, inflammation in psoriasis is not confined to skin; evidence of chronic systemic inflammation exists [10]. The systemic inflammatory milieu in psoriasis likely contributes to the atherosclerosis (considered an inflammatory disease) present in this disease [11]. Specifically, increased levels of IL-1 have been found in this systemic inflammatory milieu [12]. Since IL-1α and IL-1β have been shown to contribute to vascular inflammation and atherosclerosis [13–15], the increased levels of serum IL-1 noted in psoriasis may be responsible for promoting atherosclerosis.

Diacerein is an IL-1 inhibitor, approved in Europe as an oral anti-inflammatory treatment for osteoarthritis. The aim of this study is to investigate IL-1-driven regulation of genes related to inflammation and atherogenesis in human coronary artery endothelial cells (EC) and keratinocytes (KC), and to study the effects of diacerein on this gene regulation. ECs and KCs were utilized because they are directly involved in atherogenesis and psoriasis pathogenesis, respectively [8, 16].

## Results

### In endothelial cells treated with IL-1α

PCR-Array: mRNA expression of 16 of 84 atherosclerosis-related genes were significantly regulated at least 2-fold by IL-1α alone in comparison to control (p<.05), ranging from 8.3-fold (SELPLG) to 108.4-fold (TGFB2) up-regulation. Other genes regulated include ACE, BCL2L1, CSF2, FAS, ICAM1, LIF, MMP1, NR1H3, PLIN2, PPARG, SELE, ITGA2, TGFB1 and TNFAIP3. Adding 15µM diacerein partially or completely reversed these regulations in 15 of the 16 genes (**Table 1**). Real-time PCR verified the PCR-Array results of IL-1-induced regulation and their reversal by diacerein for ICAM1 and SELE (these and other real-time PCR data not shown).

**Table 1:** Atherosclerosis PCR-array: genes regulated by IL-1α and diacerein in ECs^1^

Refseq	Symbol	Name/description of gene	mRNA fold-regulation, with IL-1α only	mRNA fold-regulation, with IL-1α + 15μM diacerein
NM_000789	ACE	Angiotensin I converting enzyme 1	52.0382	-3.5599
NM_000633	BCL2L1	BCL2-like 1	12.9257	-1.3853
NM_000758	CSF2	Colony stimulating factor 2 (granulocyte-macrophage)	-84.2634	-7.2828
NM_000043	FAS	Fas (TNF receptor superfamily, member 6)	72.9667	5.6288
NM_000201	ICAM1	Intercellular adhesion molecule 1	39.2105	6.0664
NM_002203	ITGA2	Integrin, alpha 2 (CD49B, alpha 2 subunit of VLA-2 receptor)	8.7675	2.5589
NM_002309	LIF	Leukemia inhibitory factor (cholinergic differentiation factor)	43.1298	73.4064
NM_002421	MMP1	Matrix metallopeptidase/metalloproteinase 1 (interstitial collagenase)	22.9141	2.2828
NM_005693	NR1H3	Nuclear receptor subfamily 1, group H, member 3	13.7768	-1.8928
NM_001122	PLIN2	Perilipin 2	12.6597	-1.0402
NM_015869	PPARG	Peroxisome proliferator-activated receptor gamma	19.6415	-3.1847
NM_000450	SELE	Selectin E	15.8363	1.6804
NM_003006	SELPLG	Selectin P ligand	8.3349	-3.1635
NM_000660	TGFB1	Transforming growth factor, beta 1	28.664	2.9509
NM_003238	TGFB2	Transforming growth factor, beta 2	108.3709	6.0987
NM_006290	TNFAIP3	Tumor necrosis factor, alpha-induced protein 3	-2.1267	2.7613

1. All mRNA fold regulations are in comparison to control. These 16 genes were significantly regulated at least 2-fold by IL-1α (p<.05). 15µM -diacerein treatment reversed the effect of IL-1α in 15 genes, excepting LIF.

**Fig 1:**
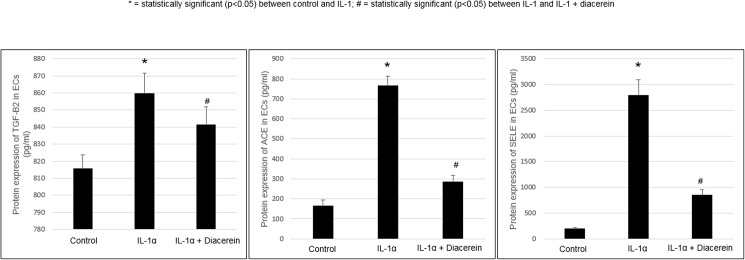
Regulation of genes at protein levels with IL-1α and diacerein in ECs. Protein expression by ELISA of TGF-B2, ACE, and SELE. Human endothelial cells were treated for 24 hours with IL-1α and diacerein as described in Methods. Cell culture supernatant was measured by ELISA assays according to the manufacturer’s instructions. Values are expressed as the mean ± SEM (*n* = 4). *P < 0.05 *vs*. control.

ELISA: quantitative protein expression of ACE, SELE and TGFB2 confirmed their up-regulation found on the mRNA level. Diacerein reversed IL-1α-induced protein up-regulation of these 3 genes. (**Fig 1**)

### In endothelial cells treated with IL-1β

PCR-Array: mRNA expression of 18 of 84 atherosclerosis-related genes were significantly regulated at least 2-fold by IL-1β alone in comparison to control (p<.05), ranging from 2.3-fold (LDLR) to 1378.9-fold (CSF2) up-regulation. Other genes regulated include BCL2A1, BIRC3, CCL2, CCL5, CD44, CSF1, ICAM1, IL1A, NFKB1, SELE, SELL, SERPINB2, TNC, TNF, TNFAIP3 and VCAM1. Adding 50µM diacerein partially or completely reversed these regulations in all 18 genes (**Table 2**)

**Table 2:** Atherosclerosis PCR-array: genes regulated by IL-1β and diacerein in ECs^1^

Refseq	Symbol	Name/description of gene	mRNA fold-regulation, with IL-1β only	mRNA fold-regulation, with IL-1β + 50μM Diacerein
NM_004049	BCL2A1	BCL2-related protein A1	13.7129	3.5032
NM_001165	BIRC3	Baculoviral IAP repeat containing 3	9.4625	2.2038
NM_002982	CCL2	Chemokine (C-C motif) ligand 2	3.8626	1.4233
NM_002985	CCL5	Chemokine (C-C motif) ligand 5	24.6279	12.0685
NM_000610	CD44	CD44 molecule (Indian blood group)	4.3224	1.9634
NM_000757	CSF1	Colony stimulating factor 1 (macrophage)	5.0849	1.4718
NM_000758	CSF2	Colony stimulating factor 2 (granulocyte-macrophage)	1378.9153	64.44
NM_000201	ICAM1	Intercellular adhesion molecule 1	9.4378	4.5319
NM_000575	IL1A	Interleukin 1, alpha	8.2156	3.0368
NM_000527	LDLR	Low density lipoprotein receptor	2.363	1.5953
NM_003998	NFKB1	Nuclear factor of kappa light polypeptide gene enhancer in B-cells 1	3.5548	1.3165
NM_000450	SELE	Selectin E	60.2549	11.0446
NM_000655	SELL	Selectin L	3.4971	-2.244
NM_002575	SERPINB2	Serpin peptidase inhibitor, clade B (ovalbumin), member 2	2.4898	1.6138
NM_002160	TNC	Tenascin C	78.3031	2.5861
NM_000594	TNF	Tumor necrosis factor	55.4908	1.3097
NM_006290	TNFAIP3	Tumor necrosis factor, alpha-induced protein 3	10.591	6.3517
NM_001078	VCAM1	Vascular cell adhesion molecule 1	5.7961	-2.1991

1. All mRNA fold regulations are in comparison to control. These 18 genes were significantly regulated at least 2-fold by IL-1β (p<.05). 50µM -diacerein treatment reversed the effect of IL-1β in all 18 genes.

**Fig 2:**
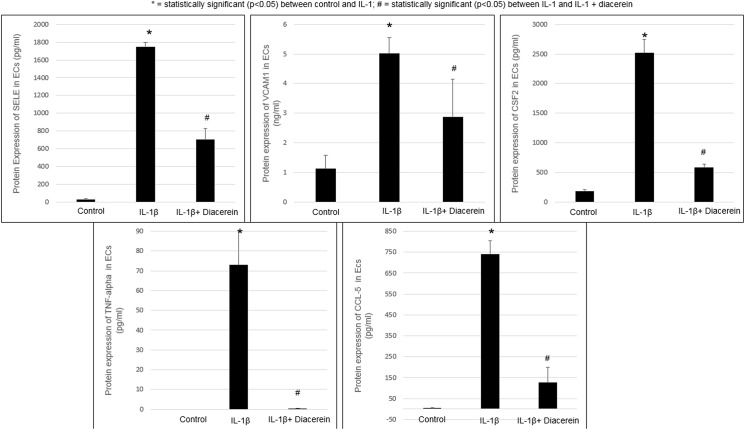
Regulation of genes at the protein level with IL-1β and diacerein in ECs. Protein expression by ELISA of SELE, VCAM1, CSF2, TNF-alpha, and CCL5. Human primary keratinocytes were treated for 24 hours with IL-1β and diacerein as described in Methods. Cell culture supernatant was measured by ELISA assays according to the manufacturer’s instructions. Values are expressed as the mean ± SEM (*n* = 4). *P < 0.05 *vs*. control.

ELISA: quantitative protein expression of SELE, VCAM1, CSF2, TNF and CCL5 confirmed their up-regulation found on the mRNA level. Diacerein reversed IL-1β-induced protein up-regulation of these 5 genes. (**Fig 2**)

### In keratinocytes treated with IL-1α

PCR-Array: mRNA expression of 24 of 370 inflammation-related genes were significantly regulated at least 2-fold by IL-1α alone in comparison to control (p<.05), ranging from 2.1-fold (CCL20) to 377.2-fold (IL-9) up-regulation. Other genes regulated include C3, CCL20, CSF2, CSF3, CXCL2, CXCL3, CXCL6, CXCR3, IL17C, IL17RB, IL23A, IL36G, IL9, ITGB2, NAMPT, NOX5, PTAFR, S100A8, SERPINA3, SPRED1, TNFSF10, and TNFSF18. Adding 10µM diacerein partially or completely reversed these regulations in all 24 genes (**Table 3**). Real-time PCR verified the PCR-Array results of IL-1-induced regulation and their reversal by diacerein for CXCL2.

**Table 3:** Inflammation PCR-array: genes regulated by IL-1α and diacerein in KCs^1^

Refseq	Symbol	Name/description of gene	mRNA fold-regulation, with IL-1α only	mRNA fold-regulation, with IL-1α + 10μM diacerein
NM_001719	BMP7	Bone morphogenetic protein 7	15.1002	2.0256
NM_000064	C3	Complement component 3	38.5144	-2.0974
NM_004591	CCL20	Chemokine (C-C motif) ligand 20	2.1197	-2.3636
NM_000758	CSF2	Colony stimulating factor 2 (granulocyte-macrophage)	3.8322	1.426
NM_000759	CSF3	Colony stimulating factor 3 (granulocyte)	5.9347	3.9513
NM_002089	CXCL2	Chemokine (C-X-C motif) ligand 2	4.9058	1.3513
NM_002090	CXCL3	Chemokine (C-X-C motif) ligand 3	4.8753	-1.0112
NM_002993	**CXCL6**	Chemokine (C-X-C motif) ligand 6 (granulocyte chemotactic protein 2)	3.5607	-5.1242
NM_001504	CXCR3	Chemokine (C-X-C motif) receptor 3	9.4556	1.5182
NM_013278	IL17C	Interleukin 17C	4.7662	-1.4756
NM_018725	IL17RB	Interleukin 17 receptor B	4.3645	1.4634
NM_016584	IL23A	Interleukin 23, alpha subunit p19	20.452	3.0674
NM_019618	IL36G	Interleukin 36, gamma	5.1964	3.0377
NM_000590	IL9	Interleukin 9	377.2329	1.9625
NM_000211	ITGB2	Integrin, beta 2 (complement component 3 receptor 3 and 4 subunit)	8.0995	5.3
NM_005746	NAMPT	Nicotinamide phosphoribosyltransferase	3.555	2.051
NM_024505	NOX5	NADPH oxidase, EF-hand calcium binding domain 5	5.3499	1.9234
NM_000952	PTAFR	Platelet-activating factor receptor	3.519	1.3278
NM_002964	S100A8	S100 calcium binding protein A8	3.5599	1.0497
NM_001085	SERPINA3	Serpin peptidase inhibitor, clade A (alpha-1 antiproteinase, antitrypsin), member 3	7.3982	3.7974
NM_152594	SPRED1	Sprouty-related, EVH1 domain containing 1	3.8884	1.8176
NM_003265	TLR3	Toll-like receptor 3	3.2652	-23.5611
NM_003810	TNFSF10	Tumor necrosis factor (ligand) superfamily, member 10	3.4818	-3.4271
NM_005092	TNFSF18	Tumor necrosis factor (ligand) superfamily, member 18	31.3306	3.9068

1. All mRNA fold regulations are in comparison to control. These 24 genes were significantly regulated at least 2-fold by IL-1α (p<.05) 10µM -diacerein treatment reversed the effect of IL-1α in all 24 genes.

**Fig 3:**
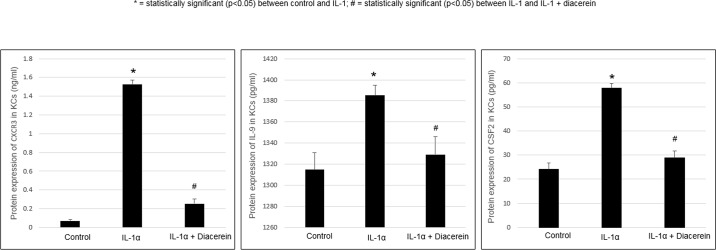
Regulation of genes at protein levels with IL-1α and diacerein in KCs. Protein expression by ELISA of CXCR3, IL-9, CSF2. Human primary keratinocytes were treated for 24 hours with IL-1α and diacerein as described in Methods. Cell culture supernatant was measured by ELISA assays according to the manufacturer’s instructions. Values are expressed as the mean ± SEM (*n* = 4). *P < 0.05 *vs*. control.

ELISA: quantitative protein expression of CXCR3, IL9 and CSF2 confirmed their up-regulation found on the mRNA level. Diacerein reversed IL-1α-induced protein regulation of these 3 genes. (**Fig 3**)

### In keratinocytes treated with IL-1β

PCR-Array: mRNA expression of 44 of 370 inflammation-related genes were significantly regulated at least 2-fold by IL-1β alone in comparison to control (p<.05) ranging from 2.1-fold (HDAC9) to 70.9-fold (S100A8) up-regulation. Other genes regulated include CCL11, 20, 28, and 5, CXCL1, 2 and 6, GDF2, HDAC9, IL32, LIF, OLR1 and SYK (partial list). Adding 20µM diacerein partially or completely reversed these regulations in all 44 genes. (**Table 4**)

**Table 4:** Inflammation PCR-array: genes regulated by IL-1β and diacerein in KCs^1^

Refseq	Symbol	Name/description of gene	mRNA fold regulation, with IL-1β only	mRNA fold regulation, with IL-1β + 20μM diacerein
NM_030882	APOL2	Apolipoprotein L, 2	2.3158	1.2406
NM_000064	C3	Complement Comp 3	8.4772	3.6511
NM_002986	CCL11	Chemokine ligand 11	2.5386	1.4083
NM_004591	CCL20	Chemokine ligand 20	9.692	8.1897
NM_148672	CCL28	Chemokine ligand 28	10.4915	3.19
NM_002985	CCL5	Chemokine ligand 5	4.0996	3.127
NM_016557	CCRL1	Chemokine receptor-like 1	2.5208	1.7298
NM_000591	CD14	CD 14 molecule	5.5239	3.715
NM_001242	CD27	CD 27 molecule	2.4294	1.3867
NM_001250	CD40	CD 40 molecule	8.0095	3.5229
NM_000759	CSF3	Colony stimulating factor 3 (granulocyte)	4.3211	3.0818
NM_001511	CXCL1	Chemokine CXC ligand 1	8.9677	1.8847
NM_002089	CXCL2	Chemokine CXC ligand 2	10.719	2.5911
NM_002993	CXCL6	Chemokine CXC 6	17.8413	1.8856
NM_005755	EBI3	Epstein-Barr virus induced-3	4.1834	2.5964
NM_000799	EPO	Erythropoietin	6.7592	-22.9238
NM_000132	F8	Coagulation factor VIII, procoagulant component	3.2593	1.7314
NM_002026	FN1	Fibronectin 1	10.7261	9.5456
NM_016204	GDF2	Growth differentiation factor 2	2.503	-1.3364
NM_000175	GPI	Glucose-6-phosphate isomerase	4.2766	3.2351
NM_013372	GREM1	Gremlin 1	4.4034	2.8852
NM_178425	HDAC9	Histone deacetylase 9	2.1274	1.5499
NM_176891	IFNE	Interferon, epsilon	5.2112	2.1267
NM_020124	IFNK	Interferon, kappa	3.2673	2.3779
NM_000576	IL1B	Interleukin 1, beta	3.9408	3.1301
NM_014432	IL20RA	Interleukin 20 receptor, alpha	4.0027	1.153
NM_004221	IL32	Interleukin 32	3.7943	1.9159
NM_019618	IL36G	Interleukin 36, alpha	4.4501	3.9432
NM_003994	KITLG	KIT ligand	7.6168	4.9655
NM_002309	LIF	Leukemia inhibitory factor	3.9508	2.1524
NM_002349	LY75	Lymphocyte antigen 75	4.1975	-1.5135
NM_002391	MDK	Midkine (neurite growth-promoting factor 2)	5.0469	3.6923
NM_002543	OLR1	Oxidized low density lipoprotein (lectin-like) receptor 1	2.5423	1.9627
NM_006404	PROCR	Protein C receptor, endothelial	5.1523	4.3059
NM_018663	PXMP2	Peroxisomal membrane protein 2, 22kDa	3.1204	2.0392
NM_002964	S100A8	S100 calcium binding protein A8	70.9305	28.5903
NM_006512	SAA4	Serum amyloid A4, constitutive	4.0531	2.4681
NM_001085	SERPINA3	Serpin peptidase inhibitor, clade A	15.5997	8.1322
NM_003177	SYK	Spleen tyrosine kinase	4.9529	3.9094
NM_003263	TLR1	Toll-like receptor 1	2.9755	1.1086
NM_003265	TLR3	Toll-like receptor 3	4.0467	1.2142
NM_006068	TLR6	Toll-like receptor 6	5.483	4.0153
NM_000594	TNF	Tumor necrosis factor	10.0188	4.1693
NM_003810	TNFSF10	Tumor necrosis factor (ligand) superfamily, member 10	18.1224	5.4619

1. All mRNA fold regulations are in comparison to control. These 44 genes were significantly regulated at least 2-fold by IL-1β (p<.05). 20µM diacerein treatment reversed the effect of IL-1β in all 44 genes.

## Discussion

Our results demonstrate that IL-1 acts as a pro-atherogenic and pro-inflammatory mediator in ECs and KCs. Addition of diacerein yielded complete or partial reversal of IL-1-induced gene regulation.

### Endothelial cells

#### Diacerein reverses IL-1α-induced regulation of atherosclerosis-related genes in ECs (Table 1)

IL-1α was found to be pro-atherogenic by regulating several genes in ECs including up-regulation of ACE, ICAM1and other genes; these regulations were reversed by diacerein, thus acting in an anti-atherogenic manner. ACE (angiotensin-1 converting enzyme) increases angiotensin-II, which elevates blood pressure. Elevated blood pressure accelerates atherogenesis [16] while angiotensin-II may independently promote vascular inflammation by increasing oxidative stress in vessel walls [17] ICAM1 (inter-cellular adhesion molecule-1) was also up-regulated by IL-1α in other studies [18, 19]. ICAM1, involved with inflammatory cell recruitment, was increased in atherosclerotic lesions [20]. Another study showed that reduced expression of cellular adhesion molecules in mice decreased atherogenicity [21]. Our IL-1α up-regulation of MMP1 (matrix metalloproteinase-1), was consistent with the findings of Hanemaaijer et al. [22] and was reversed by diacerein. MMP1 contributes to inflammatory cell intimal infiltration, plaque instability and rupture [23] and is correlated with increased total plaque burden [24]. Metalloproteinases are also implicated in multiple phases of atherosclerosis [25]. PLIN2 (perilipin-2), promoting foam-cell formation, was reported as a safe target for anti-atherogenic therapy [26]. SELE (E-selectin; facilitates vascular inflammatory cell infiltration) was up-regulated by IL-1α, consistent with Etter et al. [27]. We found diacerein significantly reduced EC protein expression of E-selectin, confirming functional down-regulation of the gene (**Fig 1**). E-selectin’s importance to atherosclerosis was demonstrated in studies showing increased E-selectin on arterial plaque surfaces [28] and reduced development of atherosclerotic lesions in mice lacking SELE [29]. Diacerein also inhibited up-regulation of SELPLG (P-selectin ligand), which helps to recruit leukocytes to the endothelium [30].

Two genes were regulated in unique ways. TNFAIP3 (TNF-alpha-induced protein-3) was down-regulated by IL-1α, in contrast to genes discussed above. However IL-1α’s effect was still pro-atherogenic because TNFAIP3 is reported to decrease inflammation and atherosclerosis in murine models [31]. Regarding LIF (leukemia inhibitory factor), two studies showed that its expression is inversely correlated with coronary atherosclerosis [32, 33]. We found IL-1α increased LIF expression, but diacerein further up-regulated the gene, enhancing LIF’s anti-atherogenic correlation.

#### Diacerein reverses IL-1β-induced regulation of atherosclerosis-related genes in ECs (Table 2)

IL-1β was found to be pro-atherogenic by regulating several genes in ECs including up-regulating CCL2, CCL5and other genes; these were reversed by diacerein, thus acting in an anti-atherogenic manner. CCL2 and CCL5 (chemokines) promote vascular inflammation by attracting leukocytes to vessel walls. Additionally, CCL2 is absent in endothelium in normal conditions but is increased in the setting of atherosclerosis and associated with elevated risk of myocardial infarction [34]. CD44 and VCAM1 were up-regulated by IL-1β, in accordance with another report of IL-1β up-regulation of VCAM1 in ECs [35]. Both CD44 and VCAM1 recruit inflammatory cells to the endothelium, while CD44 also interacts with hyaluronan, a glycosaminoglycan increased in atherosclerotic lesions. CD44 and VCAM1 were increased in an atherosclerosis mouse-model too [36]. Interestingly, we found IL-1β up-regulated IL-1α in ECs; IL-1α is reported to be a strong facilitator of atherogenesis and a prospective therapeutic target to reduce vascular inflammation [14]. Thus, diacerein may reverse IL-1α’s effects on atherosclerosis and also reduce its expression by ECs via suppressing IL-1β activity. TNC (tenascin-C), an extra-cellular matrix protein, is involved with induction of pro-inflammatory cytokines and metalloproteinases [37]. TNF (tumor necrosis factor-alpha), up-regulated by IL-1β in our study and another, [38] is thought to be pro-atherogenic by disrupting endothelial barrier function, inducing metalloproteinases and promoting vascular inflammation [39]. Diacerein reduced TNC and TNF protein expression by ECs (**Fig 2**).

#### Other genes regulated in ECs with unclear links to atherosclerosis

CSF2 (granulocyte-macrophage colony-stimulating factor; recruits inflammatory cells) was down-regulated by IL-1α but highly up-regulated by IL-1β in ECs (including increased protein expression, **Fig 2**) and up-regulated by IL-1α in KCs. Given conflicting regulation of this gene, net effects that diacerein would exert on GM-CSF activity are unclear.

Although TGFB1 (transforming growth factor-beta1) and TGFB2 (transforming growth factor-beta2) were amplified within atherosclerotic plaques in one study [40], more convincing evidence indicates these proteins actually have an athero-protective effect [41]. Diacerein decreased TGFB1 and TGFB2 mRNA and protein expression in ECs, but only to levels that remained higher than those in untreated ECs (**Fig 1**). Thus, diacerein may not have great impact on atherogenesis through TGF-beta regulation.

### Keratinocytes

**Fig 4:**
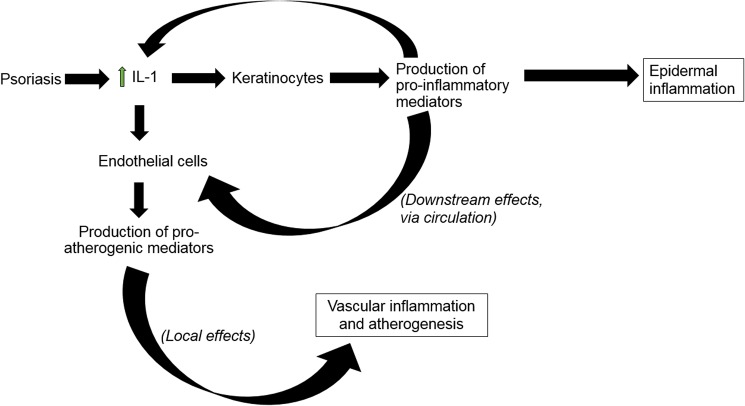
Proposed relationship of IL-1 with keratinocytes and endothelium. Increased levels of IL-1 locally in the skin and systemically in circulation lead to increase in pro-inflammatory and pro-atherogenic mediators in KCs and ECs. While these may have local effects in KCs leading to epidermal inflammation, they may also exert systemic effects by filtering into the circulation, ultimately leading to vascular inflammation downstream. The mediators produced by ECs promote atherogenesis as a local effect.

KCs are clearly involved in psoriasis pathogenesis but unlike ECs, are not directly implicated in atherogenesis. However, gene regulation observed in KCs may still have implications for atherosclerosis in the following way. It has been suggested that the skin is a source of mediators that exert not only local inflammatory effects but also enter the circulation and cause systemic inflammation [42]; as discussed previously, systemic inflammation is associated with atherosclerosis. This may be one way skin inflammation leads to systemic and vascular inflammation, ultimately contributing to atherogenesis (**Fig 4**). Also, there may be a pro-inflammatory positive feedback-loop between the epidermis and endothelium which perpetuates this effect [6]. We found various inflammatory genes regulated by IL-1 in KCs, several of which may produce mediators that have atherogenic effects downstream in the vasculature. Diacerein reversed IL-1-induced regulation of all genes in KCs, thus acting in an anti-inflammatory and potentially anti-atherogenic manner.

#### Diacerein reverses IL-1α-induced regulation of genes linked to atherosclerosis in KCs (Table 3)

IL-1α is potentially pro-atherogenic by regulating several genes in KCs; these regulations were reversed by diacerein. IL17C, a cytokine typically produced by Th17 cells, is the most plentiful IL-17 isoform in psoriatic lesions [6] and may stimulate cytokine production involved in vascular inflammation [43]. IL-23A is a cytokine that helps transform naïve CD4+ T-cells into Th17 cells, which as reported, may be pro-atherogenic [44, 45]. Serum IL-23 was also significantly elevated in patients with peripheral arterial disease, a manifestation of atherosclerosis [46]. Previous reports also showed IL-1 stimulates IL-17 and IL-23 production in T-cells [47, 48] Taken together, our data shows that IL-1α may increase Th17-mediated vascular inflammation.

IL-1α also up-regulated NOX5 in KCs, whose gene-product NADPH oxidase, stimulates production of reactive oxygen species (ROS). Dysregulation of this gene is implicated in cardiovascular disease and atherosclerosis secondary to ROS-induced vascular oxidative stress [49–51]. Zhong et al. also reported that rhein (diacerein metabolite) may protect against ROS-induced EC injury [52]. IL-9 mRNA and protein expression was considerably up-regulated by IL-1α (**Fig 3**). In a psoriasis mouse-model, IL-9 induced Th17-related inflammatory mediators [53]. Diacerein suppression of IL-9 may be anti-atherogenic by subsequently decreasing Th17-related activity. IL-1α also up-regulated NAMPT (visfatin), a pro-inflammatory cytokine elevated in psoriasis [54]. Visfatin circulating levels are correlated with clinical manifestations of atherosclerosis [55]. TNFSF18 (GITRL) is also up-regulated in KCs. Kim et al. found increased levels of GITRL’s receptor, GITR, in atherosclerotic lesions, which when activated leads to metalloproteinase and pro-inflammatory cytokine production [56]. IL-1α-induced GITRL production by KCs may cause increased binding of GITR downstream. Diacerein also down-regulates several chemokines in KCs (CCL20, CXCL2, 3, and 6), and may oppose systemic inflammation in this way.

#### Diacerein reverses IL-1β regulation of genes linked to atherosclerosis in KCs (Table 4)

IL-1β is potentially pro-atherogenic by regulating several genes in KCs; these regulations were reversed by diacerein. CSF3 (granulocyte colony-stimulating factor), up-regulated by IL-1α and IL-1β, increased endothelin-1 and decreased nitric oxide synthase in an animal model, both of which are implicated in atherogenesis [57] BMP7 (bone-morphogenetic protein-7) and GDF2 (BMP9) were up-regulated by IL-1α and IL-1β, respectively. Inhibition of BMP-signaling, as diacerein provides, may impede atherosclerosis [58]. IL-1β up-regulated HDAC9; HDACs (histone deacetylases) are involved in atherogenesis and HDAC-inhibitors are a potential therapeutic target for cardiovascular disease [59]. IL-32 also is up-regulated in KCs by IL-1β; this cytokine may induce vascular inflammation and endothelial dysfunction [60]. OLR1 (Oxidized low-density lipoprotein receptor-1) is increased in atheromatous plaques, while serum levels of ORL1 are raised in coronary artery disease patients [61]. SYK (spleen tyrosine kinase) stimulates endothelin-1, implicated in atherogenesis [62]. Correspondingly, an SYK-inhibitor decreased atherogenesis in a mouse model [63]. Other genes regulated by IL-1β in KCs with potential links to atherosclerosis include CXCL1, Ebi3, IL-36G, CCL20, CCL11, PROCR, S100A8, MDK, IL20RA, CD40, IL-1β, IL36G, TLR1, TLR6 and LY75.

Diacerein reverses IL-1-induced gene regulation in KCs. By down-regulating factors from the skin that promote and sustain inflammation, diacerein may subsequently reduce circulating levels of inflammatory mediators.

#### Diacerein and apoptosis

**Fig 5:**
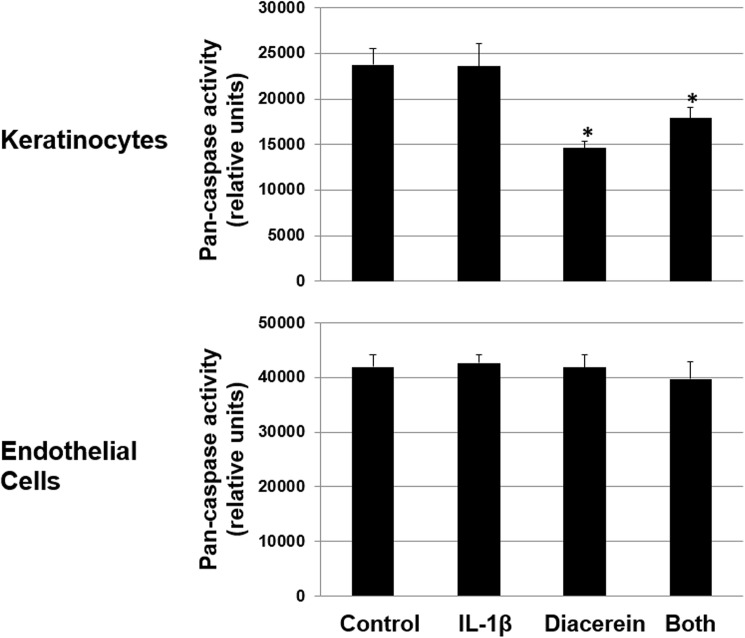
Diacerein inhibits apoptosis in keratinocytes but not in endothelial cells. Primary KCs or ECs were treated with IL-1β (10ng/ml), diacerein (20 μM for KCs and 50 μm for ECs) or both for 24 hours. Apoptosis was measured by Pan-Caspase In Situ Assay Kit as described in Methods. Values are expressed as the mean ± SEM (n = 8). * P < 0.05 vs. control.

Diacerein was reported to induce or inhibitor apoptosis in different cell types [64–66]. Interestingly, there are reports that diacerein does not affect cell apoptosis [67, 68]. In our experimental setting, we found that diacerein inhibits apoptosis in keratinocytes while it has no role in endothelial cell apoptosis (**Fig 5**). The molecular mechanisms for diacerein’s role in apoptosis of keratinocytes and endothelial cells need further investigation.

## Conclusions

Our results show that diacerein significantly opposes pro-atherogenic and pro-inflammatory effects of IL-1 on multiple genes in ECs and KCs. Psoriasis has increased IL-1 activity and increased risk of atherosclerosis and cardiovascular morbidity [3, 12]. Because our EC data and previous studies have demonstrated that IL-1α and IL-1β contribute to vascular inflammation/atherogenesis [13–15], the elevated activity of IL-1 in psoriasis may contribute to this increased risk of atherosclerosis. Additionally, our KC data show that diacerein may diminish skin inflammation but also may have potential implications for atherogenesis in psoriasis: diacerein inhibits IL-1-induced regulation of several inflammatory genes in KCs reported to have pro-atherogenic properties, downstream in the vasculature. We therefore propose a novel idea that diacerein may act to diminish IL-1-induced atherogenesis both indirectly via skin and directly on ECs in the vasculature. Interestingly, there is a recombinant, non-glycosylated antagonist of the human IL-1 receptor, anakinra. Although limited clinical data exists, one pilot study showed anakinra to have modest benefit in patients with psoriasis and psoriatic arthritis [69]. In conclusion, this study provides evidence that diacerein reverses the pro-atherogenic and pro-inflammatory gene regulation caused by IL-1 in ECs and KCs, potentially preventing progression of skin inflammation and inflammation-induced atherosclerosis. Diacerein may have an advantage over biologic therapies. Biologics like TNF-alpha inhibitors are proteins with a partial animal component and have the potential to induce an antibody response in patients, leading to the drug’s ineffectiveness. Diacerein, being a small-molecule drug administered orally, has a substantially reduced tendency to induce such antibodies. Future investigation into clinical use of diacerein to benefit both the skin and vasculature by diminishing atherogenesis and inflammation, is warranted.

## Methods

### Materials

IL-1α, IL-1β, 10% fetal-bovine-serum growth medium for KCs, penicillin/streptomycin, sodium pyruvate, non-essential amino acids (Invitrogen, Carlsbad, CA), culture medium and cell-lysis kit for ECs (Cell Applications, San Diego, CA), RNA isolation kit and protocol, First strand kit, RT^2^ SYBR Green ROX qPCR master mix, Human Inflammatory Response and Autoimmunity 384HT PCR-arrays, Human Atherosclerosis 96-well PCR-arrays (Qiagen, Valencia, CA) (Table 5) and diacerein (lyophilized powder dissolved in 50% DMSO and filtered water) (Sigma-Aldrich, St. Louis, MO) were used.

**Table 5:** List of 84 atherosclerosis-related genes and 370 inflammation-related genes tested through PCR array

**84 atherosclerosis-related genes tested** ** • Response to Stress:** ○ CCL2, CCL5, CCR1, CCR2, IL1R1, IL1R2, ITGB2, NFKB1, NOS3, SELE, SPP1, TNF, APOE, CCL5, SOD1, CCL2, CCR2, CSF2, FN1, IL4, ITGB2, TNF, CCL5, IFNAR2, TNF, CCR1, CCR2, CTGF, FN1, PDGFB, TNF, VWF, IFNG, PPARG, VEGFA. ** • Apoptosis:** ○ Anti-apoptosis: BCL2, BCL2A1, BCL2L1, BIRC3, CCL2, CFLAR, FAS (TNFRSF6), IL1A, IL2, NFKB1, SERPINB2, SPP1, TGFB1, TNF, TNFAIP3. ○ Induction of Apoptosis: APOE, BAX, BID, CFLAR, FAS (TNFRSF6). ○ Other Genes Related to Apoptosis: IL5, ITGB2. ** • Blood Coagulation and Circulation:** ○ Blood Coagulation: FGA, ITGA2, LPA, SERPINE1. ○ Circulation: APOA1, APOB, APOE, COL3A1, ELN, ENG, LPA, LPL, NPY. ○ Platelet Activation: PDGFA, PDGFB, PDGFRB, VWF. ○ Regulation of Blood Pressure: ACE, FGA. ** • Adhesion Molecules:** ○ Cell-cell Adhesion: CD44, CDH5, ICAM1, ITGB2, SELE, SELL, TNF, VCAM1, VEGFA. ○ Cell-matrix Adhesion: CD44, ITGA2, ITGA5, ITGAX, ITGB2, SPP1. ○ Other Genes Involved in Adhesion: CCL2, CCL5, CCR1, CTGF, ELN, ENG, FN1, LAMA1, SELPLG, THBS4, TNC, VWF. ** • Extracellular Molecules:** ○ ECM Protease Inhibitors: LPA, SERPINB2, SERPINE1. ○ ECM Proteases: ACE, MMP1, MMP3. ○ Extracellular Matrix (ECM) Structural Constituents: COL3A1, ELN, FN1. ○ Other Extracellular Molecules: ADFP, APOA1, APOB, APOE, CCL2, CCL5, CSF2, CTGF, FGA, FGF2, HBEGF (DTR), IFNAR2, IFNG, IL1A, IL2, IL3, IL4, IL5, LAMA1, LIF, LPL, NPY, PDGFA, PDGFB, SPP1, THBS4, TNC, VEGFA, VWF. ** • Lipid Transport and Metabolism:** ○ Cholesterol Metabolism: ABCA1, APOA1, APOB, APOE, IL4, LDLR. ○ Fatty Acid Metabolism: FABP3, LPL, PPARA, PTGS1. ○ Lipid Transport: ABCA1, APOA1, APOB, APOE, FABP3, LDLR, LPA, LPL, MSR1. ○ Lipoprotein Metabolism: APOA1, APOE, LDLR, LPL. ○ Steroid Metabolism: NR1H3, PPARA, PPARD, PPARG, RXRA. ○ Other Genes Related to Lipid Metabolism: PLIN2, APOE, LPA. ** • Cell Growth and Proliferation:** ○ Growth Factors and Receptors: CSF2, KDR, PDGFRB, SPP1. ○ Negative Regulation of Cell Proliferation: BCL2, FABP3, IL1A. ○ Positive Regulation of Cell Proliferation: CSF1, FGA, FGF2, HBEGF (DTR), IL2, IL3, IL5, LIF, VEGFA. ○ Regulation of the Cell Cycle: FGF2, IL1A, PDGFA, PDGFB, TGFB1, TGFB2, VEGFA. ○ Other Genes Involved in Cell Growth and Proliferation: CTGF, ELN, IFNG, IL4, NPY. ** • Transcription Regulators:** ○ Nuclear Receptors: NR1H3, PPARA, PPARD, PPARG, RXRA. ○ Other Transcription Regulators: EGR1, KLF2, NFKB1, TNF, TNFAIP3.
**370 inflammation-related genes tested** ** • Cytokines:** ○ Chemokines: CCL1, CCL11, CCL13, CCL16, CCL17, CCL18, CCL19, CCL2, CCL20, CCL21, CCL22, CCL23, CCL24, CCL25, CCL26, CCL27, CCL28, CCL3, CCL4, CCL5, CCL7, CCL8, CKLF, CX3CL1, CXCL1, CXCL10, CXCL11, CXCL12, CXCL13, CXCL14, CXCL2, CXCL3, CXCL5, CXCL6, CXCL9, CYP26B1, PF4V1, PPBP, PXMP2, XCL1. ○ Interleukins: IL10, IL11, IL12A, IL12B, IL13, IL15, IL16, IL17A, IL17B, IL17C, IL17D, IL17F, IL18, IL19, IL1A, IL1B, IL1F10, IL36RN, IL36A, IL37, IL36B, IL36G, IL1RN, IL2, IL20, IL21, IL22, IL23A, IL24, IL25 (IL17E), IL26, IL27, IFNL1, IL3, IL32, IL4, IL5, IL6, IL7, IL8, IL9. ○ Other Cytokines: AREG, BMP1, BMP2, BMP3, BMP7, CD40LG (TNFSF5), CD70 (TNFSF7), CLC, CMTM1 (CKLFSF1), CMTM2 (CKLFSF2), CSF1 (MCSF), CSF2 (GM-CSF), CSF3 (GCSF), CTF1, CXCL16, EBI3, EDA, EPO, FASLG, FGF1, FGF10, FGF12, FGF2 (bFGF), FGF7, FIGF, FLT3LG, GDF2, GDF3, GDF5, GDF6, GDF9, GLMN, GPI, GREM1, GREM2, GRN, IFNA1, IFNA14, IFNA2, IFNA4, IFNA8, IFNB1, IFNE, IFNG, IFNK, IFNW1, IFNWP2, IK, INHA, INHBA, INHBB, KITLG, LEFTY1, LEFTY2, LIF, LTA, LTB, MDK, MIF, MSTN, NODAL, OSM, NAMPT, PDGFA, PDGFB, PRL, PTN, SECTM1, SLURP1, SOCS2, SPP1, THPO, TNF, TNFRSF11B, TNFSF10, TNFSF11, TNFSF13, TNFSF13B, TNFSF14, TNFSF15, TNFSF18, TNFSF4, TNFSF8, TNFSF9, TRAP1, TYMP, VEGFA, VEGFB, YARS. ** • Cytokine Receptors:** ○ Chemokine Receptors: CCR1, CCR10, CCR2, CCR3, CCR4, CCR5, CCR6, CCR7, CCR8, CCR9, ACKR4, CCRL2, CX3CR1, CXCR1 (IL8RA), CXCR2 (IL8RB), CXCR3, CXCR4, CXCR5, CXCR6, XCR1. ○ Interleukin Receptors: IL10RA, IL10RB, IL11RA, IL12B, IL12RB1, IL12RB2, IL13RA1, IL13RA2, IL15RA, IL17RA, IL17RB, IL18R1, IL1R1, IL1R2, IL1RAP, IL1RAPL2, IL1RL1, IL1RL2, IL20RA, IL21R, IL22RA1, IL22RA2, IFNLR1, IL2RA, IL2RB, IL2RG, IL31RA, IL3RA, IL4R, IL5RA, IL6R, IL6ST, IL7R, IL9R ○ Other Cytokine Receptors: CNTFR, CSF2RA, CSF2RB, CSF3R, EBI3, EPOR, F3, GFRA1, GFRA2, GHR, IFNAR1, IFNAR2, IFNGR1, IFNGR2, LEPR, LIFR, MPL, OSMR, PRLR, TTN. ** • Cytokine Metabolism:** ○ APOA2, AZU1, CD27 (TNFRSF7), CD28, CD4, CD86, EBI3, GLMN, IL10, IL12B, IL17F, IL18, IL21, IL27, IL4, INHA, INHBA, INHBB, IRF4, NLRP12, PRG3, S100B, SFTPD, SIGIRR, TLR1, TLR3, TLR4, TLR6, TNFSF15. ** • Cytokine Production:** ○ APOA2, AZU1, CD27 (TNFRSF7), CD28, CD4, CD86, EBI3, GLMN, IL10, IL12B, IL17F, IL18, IL21, IL27, IL4, INHA, INHBA, INHBB, INS, IRF4, NFAM1, NLRP12, NOX5, PRG3, S100B, SFTPD, SIGIRR, TLR1, TLR3, TLR4, TLR6. ** • Cytokine-Cytokine Receptor Interaction:** ○ CCR1, CD40 (TNFRSF5), CXCR3, IL18RAP, IL23R, XCR1. ** • Acute-Phase Response:** ○ AHSG, APCS, APOL2, CEBPB, CRP, F2, F8, FN1, IL22, IL6, INS, ITIH4, LBP, REG3A (PAP), REG3G, SAA4, SERPINA1, SERPINA3, SERPINF2, SIGIRR, STAT3. ** • Inflammatory Response:** ○ AIMP1, ADORA1, AIF1, APOA2, APOL3, AZU1, BCL6, BLNK, C3, C3AR1, CCL1, CCL11, CCL13, CCL16, CCL17, CCL18, CCL19, CCL2, CCL20, CCL21, CCL22, CCL23, CCL24, CCL25, CCL26, CCL3, CCL4, CCL5, CCL7, CCL8, CCR1, CCR2, CCR3, CCR4, CCR7, CD14, CD180, CD40, CD40LG, CD74, CD97, CKLF, CX3CL1, CXCL1, CXCL10, CXCL11, CXCL12, CXCL13, CXCL14, CXCL16, CXCL2, CXCL3, CXCL5, CXCL6, CXCL9, CXCR1 (IL8RA), CXCR2 (IL8RB), CYBB, DOCK2, EPHX2, F11R, FOS, FPR1, GPR68, HDAC4, HDAC5, HDAC7, HDAC9, HRH1, CARD18, IFNA2, IL10, IL10RB, IL13, IL17A, IL17B, IL17C, IL17D, IL17F, IL18RAP, IL1A, IL1B, IL1F10, IL36RN, IL36A, IL1R1, IL1RAP, IL1RN, IL20, IL25 (IL17E), IL31RA, IL5, IL8, IL9, IRF7, ITGB2, KNG1, LTB4R, LY75, LY86, LY96, MEFV, MGLL, MIF, MMP25, MYD88, NCR3, NFAM1, NFATC3, NFATC4, NFE2L1, NFKB1, NFRKB, NFX1, NLRP12, NMI, NOS2 (iNOS), NR3C1, OLR1, PARP4, PGLYRP1, PLA2G2D, PLA2G7, PRDX5, PREX1, PRG2, PRG3, PROCR, PROK2, PTAFR, PTGS2 (COX2), PTPRA, PTX3, RIPK2, S100A12, S100A8, SCUBE1, SELE, SFTPD, SIGLEC1 (SN), SPACA3, SPP1, STAB1, SYK, TACR1, TIRAP, TLR1, TLR10, TLR2, TLR3, TLR4, TLR5, TLR6, TLR7, TLR8, TLR9, TNF, TNFAIP6, TOLLIP, TPST1, VPS45, XCR1. ** • Humoral Immune Response:** ○ BLNK, C3, CCL16, CCL18, CCL2, CCL20, CCL22, CCL3, CCL7, CCR2, CCR6, CCR7, CCRL2, CD27 (TNFRSF7), CD28, CD40, CD74, CD86, CLC, CSF2RB, CXCR3, CYBB, EBI3, GPI, IL10, IL12A, IL12B, IL12RB1, IL13, IL18, IL1B, IL2, IL26, IL4, IL6, IL7, IL7R, IRF4, ITGB2, LY86, LY96, NFKB1, PTAFR, S100B, SFTPD, XCL1, XCR1. ** • Other Genes Involved in Immune Response:** ○ CAST, ERBB2, ERBB2IP, MUC4, SDCBP, SLCO1A2, SPRED1, SRGAP1.

### Cell culture and treatment

Human primary keratinocytes (Invitrogen, Carlsbad, CA, catalog# C-005-25P-A) and human primary coronary artery endothelial cells (Cell Applications Inc., San Diego, CA, catalog# 300K-05a) were cultured under standard conditions (humidified atmosphere, 5% CO2 at 37°C) in growth medium supplemented with sodium pyruvate, non-essential amino acids and penicillin/streptomycin; media were replaced every 48 hours. KCs were differentiated from basal to mature keratinocytes by placing into a higher-calcium medium for 48–72 hours. At 80% confluence, cells were treated for 24 hours either with IL-1α or IL-1β only (25ng/ml IL-1α or 10ng/ml IL-1β in KCs and 10ng/ml IL-1α or 10ng/ml IL-1β in ECs), IL-1α or IL-1β combined with diacerein (diacerein concentrations: 10μM in KCs and 15μM in ECs with IL-1α; 20μM in KCs and 50μM in ECs with IL-1β–these concentrations were established using a dose-response curve prior to the experiment), or vehicle (DMSO). After 24 hours, cell culture supernatant was collected and stored at -80°C, and cells were washed with ice-cold phosphate-buffered-saline and frozen at -80°C until RNA isolation.

### RNA isolation and real-time polymerase chain reaction (PCR) analysis

Total RNA from KCs and ECs were isolated using Trizol reagent per manufacturer’s instructions. Reverse-transcription using First strand kit formed cDNA. Genes reported in the literature to be regulated by IL-1 and relevant to the pathogenesis of skin or vascular inflammation/atherosclerosis, were tested by real-time PCR using custom primers (Integrated DNA Technologies, Coralville, IA).

### PCR-array

Using KC and EC cDNA, PCR-arrays were used per manufacturer’s instructions. 10μL of master mix with cDNA was loaded into each well, and PCR reaction was run (ViiA-7 PCR machine, Applied Biosystems, Waltham, MA). Cycling conditions used: 95°C (10 minutes), then 40 repeating cycles of 95°C (15 seconds) followed by 60°C (60 seconds). Experiments were repeated in triplicate using four biologic replicates. Raw data were analyzed by a web-based Array Analysis Software (Qiagen). Data was normalized using house-keeping genes (ACTB, B2M, GAPDH, HPRT1, RPLP0). We then identified genes that were at least 2-fold up- or down-regulated after addition of IL-1 and/or diacerein in comparison to control, with p-value<0.05.

### Enzyme linked immunosorbent assay (ELISA)

ELISA-kits were purchased based on PCR-array result. Kit were used according to manufacturer’s instructions. ELISA micro-plates were analyzed using Synergy HT Multi-Mode Microplate Reader (BioTek, Winooski, VT) per the kit protocol, usually 450nm.

For IL-1α-treated cells, CSF2 (Sigma-Aldrich), SELE (Sigma-Aldrich), IL-9 (Sigma-Aldrich), CXCR3 (MyBioSource.com, San Diego, CA), TGF-B2 (Abcam, Cambridge, MA) and ACE (Abcam) human ELISA-kits were used.

For IL-1β-treated cells, E-Selectin (Sigma-Aldrich), TNF-alpha (Invitrogen) CCL5 (Biomatic USCN Life Sciences Inc., Wuhan, Hubei, China) VCAM-1 (Sigma-Aldrich) and CSF2 (Sigma-Aldrich) human ELISA-kits were used.

### Pan-caspase assay

Pan-caspase assay was performed using CaspaTag™ Pan-Caspase In Situ Assay Kit (EMD Millipore, Billerica, MA) according to the manufacturer’s instructions. Briefly, cells were seeded in 12-well plates and harvested 24 hours after treatment. Cells were trypsinized and centrifuged after trypsin inactivation. Next 90 μl of 1% charcoal-dextran-treated FBS was added into each tube with 3 μl of 30X FLICA to resuspend cells. Then cells were incubated for 1 hour at 37°C under 5% CO2. After a series of washes, cells were resuspended in PBS and counted. Absorbance using an excitation wavelength of 485 nm and an emission wavelength of 520 nm was read in a Tecan Infinite 200 Pro multimode reader (Tecan US Inc., Morrisville, NC).

### Statistical analysis

PCR-array raw data were analyzed as listed above. Other data reported in this study (ELISA, real-time PCR) are the mean of 4 biologic replicates +/- standard error of the mean and were analyzed by ANOVA with p-value <0.05 taken as significant.

## Supporting information

S1 File This includes original PCR array data from endothelial cells. (XLS)

S2 File This includes original PCR array data from keratinocytes. (XLS)

## Abbreviations

IL-1α: interleukin-1alpha
IL-1β: interleukin-1beta
EC: primary human coronary artery endothelial cell
KC: primary human keratinocyte
